# Clinical patterns and the evolution of relapsing polychondritis based on organ involvement: a Chinese retrospective cohort study

**DOI:** 10.1186/s13023-021-01861-x

**Published:** 2021-05-17

**Authors:** Lei Zhang, Shuang Yun, Tiange Wu, Yujie He, Jinyan Guo, Lishuai Han, Jiameng Lu, Xiaojun Liu, Rui Yang, Shitao Zhang, Tianfang Li, Shengyun Liu

**Affiliations:** 1 Rheumatology Department, The First Affiliated Hospital of Zhengzhou University, E1 Jianshe Road, Zhengzhou, 450052 Henan China; 2 Ophthalmology Department, The First Affiliated Hospital of Zhengzhou University, Zhengzhou, Henan China; 3 Pulmonology Department, The First Affiliated Hospital of Zhengzhou University, Zhengzhou, Henan China; 4 Otolaryngology Department, The First Affiliated Hospital of Zhengzhou University, Zhengzhou, Henan China

**Keywords:** Relapsing polychondritis, RPC, Classification, Clinical patterns, Diagnosis

## Abstract

**Background:**

Relapsing polychondritis (RPC) is a rare autoimmune disease and its early diagnosis remains challenging. Defining the clinical patterns and disease course may help early recognition of RPC.

**Results:**

Sixty-six males and 60 females were included in this study. The average age at onset were 47.1 ± 13.8 years and the median follow-up period was 18 months. Correlation analysis revealed a strong negative correlation between airway involvement and auricular chondritis (r = − 0.75, P < 0.001). Four distinct clinical patterns were identified: Ear pattern (50.8%), Airway pattern (38.9%), Overlap pattern (4.8%) and Airway-Ear negative pattern (5.6%), and patients with Ear pattern and Airway pattern were further divided into limited and systemic form of RPC (27.8% with limited form of Ear pattern and 24.6% with limited form of Airway pattern initially). During follow-up, a minority of patients with Ear pattern and Airway pattern progressed into Overlap pattern, and some Airway-Ear negative pattern patients progressed into Ear pattern. While a large majority of limited RPC patients remained limited form during follow-up, a minority of limited RPC patients progressed into systemic form. Patients with Ear pattern had the highest survival rate and relatively lower inflammatory status.

**Conclusions:**

RPC patients can be categorized as 4 different clinical patterns and 2 distinct presenting forms (limited and systemic) based on organ involvement. The clinical patterns and presenting forms may evolve during follow-up. Our findings may facilitate early recognition of this rare disease.

## Background

Relapsing Polychondritis (RPC) is a systemic inflammatory disorder of unknown etiology and characterized by recurrent and progressive inflammation of the cartilaginous structures, particularly involving the auricles, nose and respiratory tract as well as extra-cartilaginous tissues, including eyes, heart, skin, central nervous and hematological systems [1–3].

The incidence of RPC is about 3.5 per million per year in the U.S. [4] and 0.71 per million population per year between 1990 and 2012 in the UK [5]. Often time, patients’ first visits are to non-rheumatologic specialists [5–8], due to protean manifestations and wax and wane disease courses of RPC, which may lead to misdiagnosis and delayed diagnosis [5–13].

Because of the rarity, the diagnosis of RPC is mainly based on empirical criteria proposed by McAdams [14], Damiani [15], Michet [16] and their colleagues. However, early and prompt diagnosis is difficult to establish because of the inadequacy of clinical manifestations [8]. Meanwhile, partial RPC has been reported by Mathew et al. [11] as well as in our previous study [6]. Emerging data suggest that limited RPC are not uncommon in clinical practice [6, 17–24]. Furthermore, lack of vigilance to RPC, particularly in non-rheumatologic specialists, may contributes to diagnostic delay and misdiagnosis [6, 25, 26]. Thus, the analysis clinical patterns of RPC patients, particularly those in early stage, as well as partial and limited RPC, may help non-rheumatologic specialists get a clear and complete scenario of this rare disease, facilitating early recognition. While endeavors have been made by a few groups, these studies did not cover all clinical spectrum of this disease because they included neither patients in early stage nor those with partial or limited disease [27–29]. Therefore, our current study aimed to clarify distinct clinical patterns based on organ involvement using our own cohort including early-stage PRC patients as well as partial and limited RPC patients. In addition, we described the evolution of clinical patterns for the first time.

## Results

### Patient characteristics

This study included 66 male and 60 female patients with an average age of 47.1 ± 13.8 years at disease onset. The age of male patients was comparable to female patients at disease onset (46.1 ± 13.7 vs. 48.1 ± 13.8, P = 0.408). Median follow-up period after diagnosis was 18 months (1–153 months). Among them, 34 patients were followed up for at least 36 months, 51 for at least 24 months and 82 for at least 12 months. One patient with airway involvement was lost after follow-up for 2 months. Median disease duration since the disease onset to the last follow-up was 26 months (3–162 months). Median diagnostic delay between the appearance of the first symptom and the establishment of diagnosis was 5 months (0–132 months), and 31 patients were delayed for over a year.

### Initial and cumulative features

The most frequent initial features included auricular chondritis (n = 70,55.6%) and airway involvement (n = 55, 43.7%) (including 17 patients[13.5%] with laryngeal involvement and 48 patients[38.1%] with tracheobronchial involvement), ocular inflammation (n = 25, 19.8%), fever (n = 20,15.9%), nasal chondritis (n = 15,14.3%), arthritis (n = 16,12.7%), hearing loss (n = 12,9.5%), cardiac involvement (n = 8, 6.3%), costochondritis (n = 3, 2.4%), neurological involvement (n = 6, 4.8%), cutaneous lesions (n = 1, 0.8%), and myelodysplastic syndrome (n = 1, 0.8%). More cumulative features developed during follow-up (Table 1). Cumulative features in previous reports were also detailed in Table 1 [7–9, 11, 13, 14, 16, 27, 30, 31].

**Table 1:** Characteristics of patients with relapsing polychondritis in present study and previous series

Clinical features	Present study	Ferrada et al., 2018 [8]	Dion et al., 2016 [27]	Lin DF et al., 2016^a^ [7]	Oka H et al., 2014 [30]	Mathew et al., 2012 [11]	Trentham et al.,1998 [13]	Zeuner et al., 1997 [9]	Michet et al., 1986[16]	McAdam et al., 1976^b^ [14]
No. of patients	126	304	142	158	239	43	66	62	112	159
Country	North China	International	France	South China	Japan	USA	USA	German	USA	USA
Study design	Retro	Survey	Retro	Retro	Survey	Retro	Retro	Survey	Retro	Retro + Liter
Demographic characteristics										
Sex, F/M	60/66	263/41	86/56	65/93	112/127	23/20	49/17	26/36	55/57	76/83
Mean age at onset, years	47.1	NA	43.5	NA	52.7	NA	NA	NA	NA	NA
Mean age at diagnosis, years	47.8	43.2	NA	45.3	NA	43	46	46.6*	51*	43.8
Follow-up, months	18 (1–153)	NA	156!	NA	NA	NA	96!	11!	72 (1–240)	4.3 !
Median delay, months	5 (0–132)	NA	12 (0–300)	14 (0.3–168)	NA	38.4**	34.8**	NA	NA	NA
Clinical features, n (%)										
Auricular chondritis	75 (59.6)	262 (86.2)	127 (89.4)	107 (67.7)	187 (78.2)	38 (88.4)	62 (93.9)	58 (93.5)	95 (84.8)	141 (88.7)
Laryngotracheal	61 (48.4)¶	NA	71 (50.0)§	109 (68.9)	119 (49.8)‡	16 (37.2)	44 (66.7)	19 (30.6)	53 (47.3)	89 (55.9)
Laryngeal	20 (15.9)	180 (59.2)	61 (43.0)	45/128 (35.2)†	47 (19.7)	NA	NA	NA	19 (16.9)	NA
Tracheobronchial	52 (41.3)	225 (74.0)	32 (22.5)	NA	97 (40.6)	NA	NA	NA	NA	NA
Ocular inflammation	34 (26.9)	151 (49.7)	80 (56.3)	70 (44.3)	109 (45.6)	23 (53.5)	37 (56.1)	31 (50.0)	57 (50.9)	104 (65.4)
Fever	26 (20.7)	NA	43 (30.3)	NA	NA	NA	NA	15 (24.2)	44 (39.3)	NA
Nasal chondritis	22 (17.5)	193 (63.5)	89 (62.7)	85 (53.8)	94 (39.3)	15 (34.8)	32 (48.5)	35 (56.5)	60 (53.6)	115 (72.3)
Arthritis	23 (18.3)	250 (82.2)	47 (33.1)	88 (55.7)	92 (38.5)	26 (60.5)	56 (84.8)	33 (53.2)	58 (51.8)	121 (76.1)
Hearing loss	15 (11.9)	104 (34.2)	39 (27.5)	39 (24.7)	52 (21.8)	16 (37.2)	28 (42.4)	12 (19.4)	29 (25.9)	65 (40.9)
Costochondritis	7 (5.6)	143 (47.0)	57 (40.1)	23 (14.6)	NA	NA	NA	NA	NA	58 (36.5)
Renal involvement	0 (0)	NA	0 (0)	4/134 (2.5)†	16 (6.7)	1 (2.3)	NA	4 (6.5)	16 (14.3)	NA
Neurological involvement	6 (4.8)	NA	17 (11.9)	8/69 (11.6)	23 (9.6)	NA	NA	6 (9.7)	NA	5 (3.1)
Skin involvement	2 (1.6)	NA	40 (28.2)	17/37 (45.9)†	32 (13.4)	NA	25 (37.9)	15 (24.2)	31 (27.7)	26 (16.4)
Valvulopathy	1 (0.8)	NA	31 (21.8)	3/98 (3.1)†	5 (2.1)^	NA	5 (7.6)	0 (0)	7 (6.3)	14 (8.8)
Arrhythmia	13 (10.3)	NA	13 (9.2)	1/98 (1.0)†	0 (0)^	NA	NA	2 (3.2)	NA	NA
MDS	1 (0.8)	NA	12 (8.2)	0 (0)	4 (1.7)	NA	NA	4 (6.5)	NA	NA
Autoimmune diseases	4 (3.2%)	NA	31 (21.8)	17 (10.8)	11 (4.6)	11 (25.6)	13 (19.7)	22 (35.5)	38 (33.9)	NA

Data are presented as n (%).Retro, retrospective; Liter, literature review; NA, not available

^a^A retrospective review of RPC patients in one medical center in south China and cases reported in Chinese literatures

^b^Among 159 patients, 23 were followed up in the author’s center, the remaining 136 patients were cited from literature

^*^Median age at diagnosis

^**^Mean delay

!Mean follow-up duration

^#^Sixty-four percent presented symptoms for more than 5 years before diagnosis

^¶^Laryngotracheal involvement was seen in 61 patients (48.4%), indicating 11 patients presented both laryngeal and tracheobronchial involvement

^§^Laryngotracheal involvement was seen in 71 patients (50%), indicating 22 patients presented both laryngeal and tracheobronchial involvement

^‡^Laryngotracheal involvement in 119 patients (50%), indicating 35 patients presented both laryngeal and tracheobronchial

^†^Denominator represents the total number in the category where datas were missing

^Data were extracted from another study conducted by the same authors [31]

At the final stage of the follow-ups, airway involvement was found in 61patients (48.4%). Among them, 20 patients (15.9%) had laryngeal involvement and 52 patients (41.3%) had tracheobronchial involvement, indicating 11 of them had both laryngeal and tracheobronchial involvement (Table 1).

Neurological involvement was seen in 6 patients (4.8%), and no new onset of neurological impairment was observed during follow-up. One patient with rapture of intracranial aneurysm (anterior communicating artery) received surgical procedures. Four patients had psychiatric symptoms including persecutory delusion, mania, hallucinations, cognitive disorder, and impaired memory, and 1 patient had headache and diplopia. Magnetic Resonance Imaging (MRI) of these latter 5 patients revealed ischemia and edema of the brain in 4 patients and demyelination in 1 patient. The lesions were detected in frontal, temporal and parietal cerebrum as well as basal ganglia and thalamus, unilaterally or bilaterally. Cerebrospinal fluid tests of these 5 patients were normal in 4 patients, but with increased white blood cells (mainly neutrophils) in 1 patient. The cerebrospinal fluid pressure increased in 2 patients and was normal in 3 patients. Cardiac involvement was found in 13 patients (10.3%), including premature contraction (n = 8), atrial tachycardia (n = 2), conduction block (n = 2), atrial fibrillation (n = 2), pre-excitation syndrome (n = 1) and valvular insufficiency (n = 1).

Associated autoimmune rheumatic conditions were found in 4 patients, including 2 patients with recurrent oral ulceration resembling Bechet’s syndrome, 1 with Sjögren's syndrome and 1 with IgG4 related disease.

### Clinical pattern and disease evolution

**Fig. 1:**
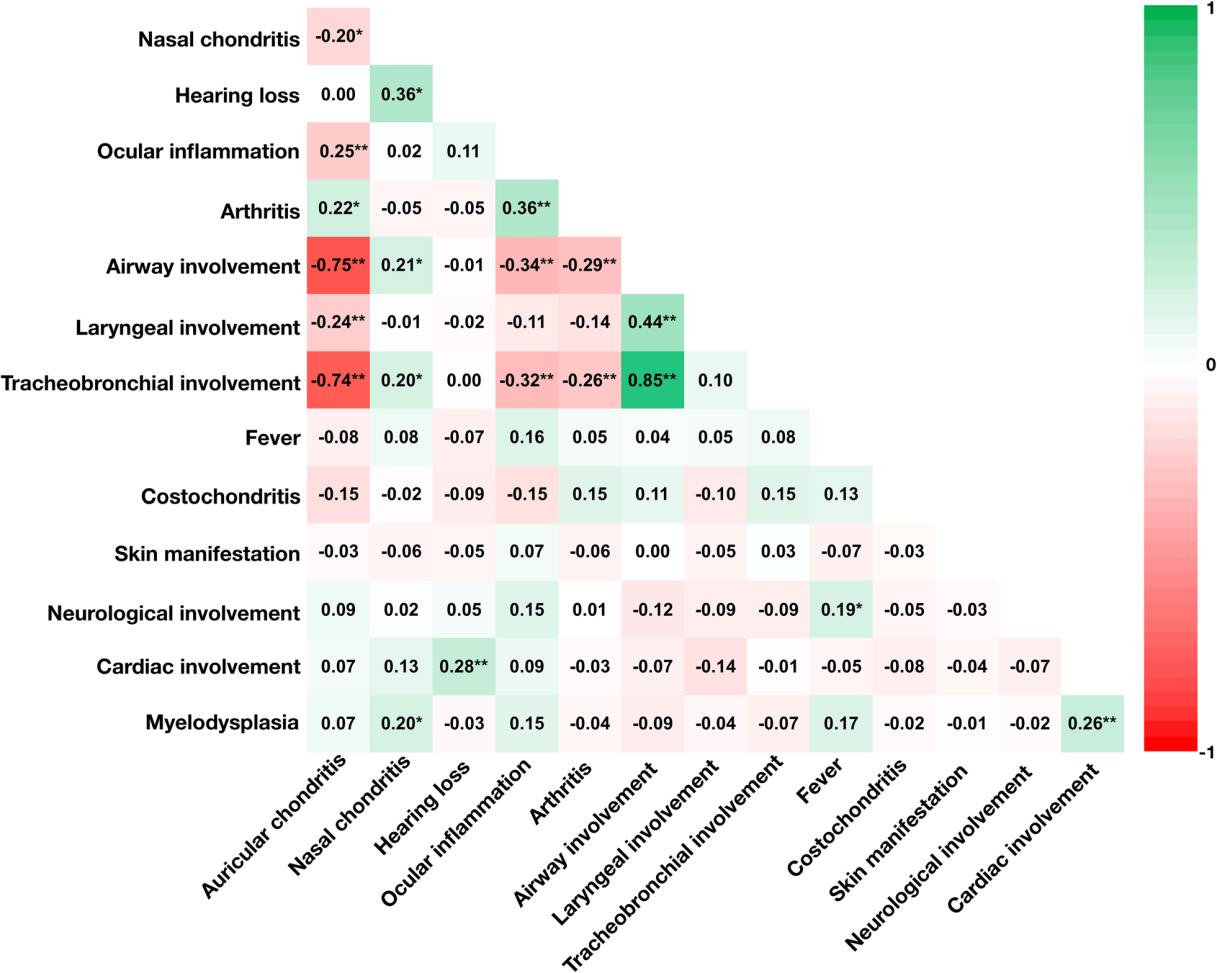
Correlation analysis of different organ involvement. The color depth is proportional to correlation strength, and red represent negative correlation while green represent positive correlation. * P < 0.05; ** P < 0.01

We performed correlation analysis and calculated correlation coefficients between cumulative organ involvement and found a strong negative correlation between airway involvement and auricular chondritis (r = -0.75, P < 0.001), and also between tracheobronchial involvement and auricular chondritis (r = -0.74, P < 0.001). We only found a weak negative correlation between ocular inflammation and airway or tracheobronchial involvement (r = − 0.34, P < 0.001 and r = − 0.32, P < 0.001 respectively). A weak positive correlation was also revealed between hearing loss and nasal chondritis (r = 0.36, P < 0.001), and between ocular inflammation and arthritis (r = 0.36, P < 0.001) (Fig. 1).

Based on these findings, we consider auricular chondritis and airway involvement to be the most distinguishable variables to define subgroups of RPC, similar to previous reports [28, 29]. Thus 4 clinical patterns were identified: Ear pattern (ear lesion without airway involvement, subgroup A), Airway pattern (airway lesion without ear involvement, subgroup B), Overlap pattern (both ear and airway involved, subgroup C) and Airway-Ear negative pattern (nether auricular nor airway involved, subgroup D) (Table 2). Apparently, a large majority of the patients were classified as Ear pattern and Airway pattern (50.8% and 38.9%, respectively at disease onset, and 49.2% and 38.1%, respectively during follow-up).

**Table 2:** Classification based on organ involvement pattern

	Organ involvement		At disease onset		During follow-up	
	Ear	Airway	Limited	Systemic	Limited	Systemic
Subgroup A	+	−	35 (27.8)	29 (23.0)	25 (19.8)	37 (29.4)
Subgroup B	−	+	31 (24.6)	18 (14.3)	26 (20.6)	22 (17.5)
Subgroup C	+	+	0 (0)	6 (4.8)	0 (0)	13 (10.3)
Subgroup D	−	−	0 (0)	7 (5.6)	0 (0)	3 (2.4)

Data are presented as n (%)

A proportion of patients were referred to as the limited RPC at disease onset as well as during the whole disease process, for they presented with auricular chondritis or airway involvement as the sole manifestation, while the rest were referred to as systemic RPC (Table 2).

**Fig. 2:**
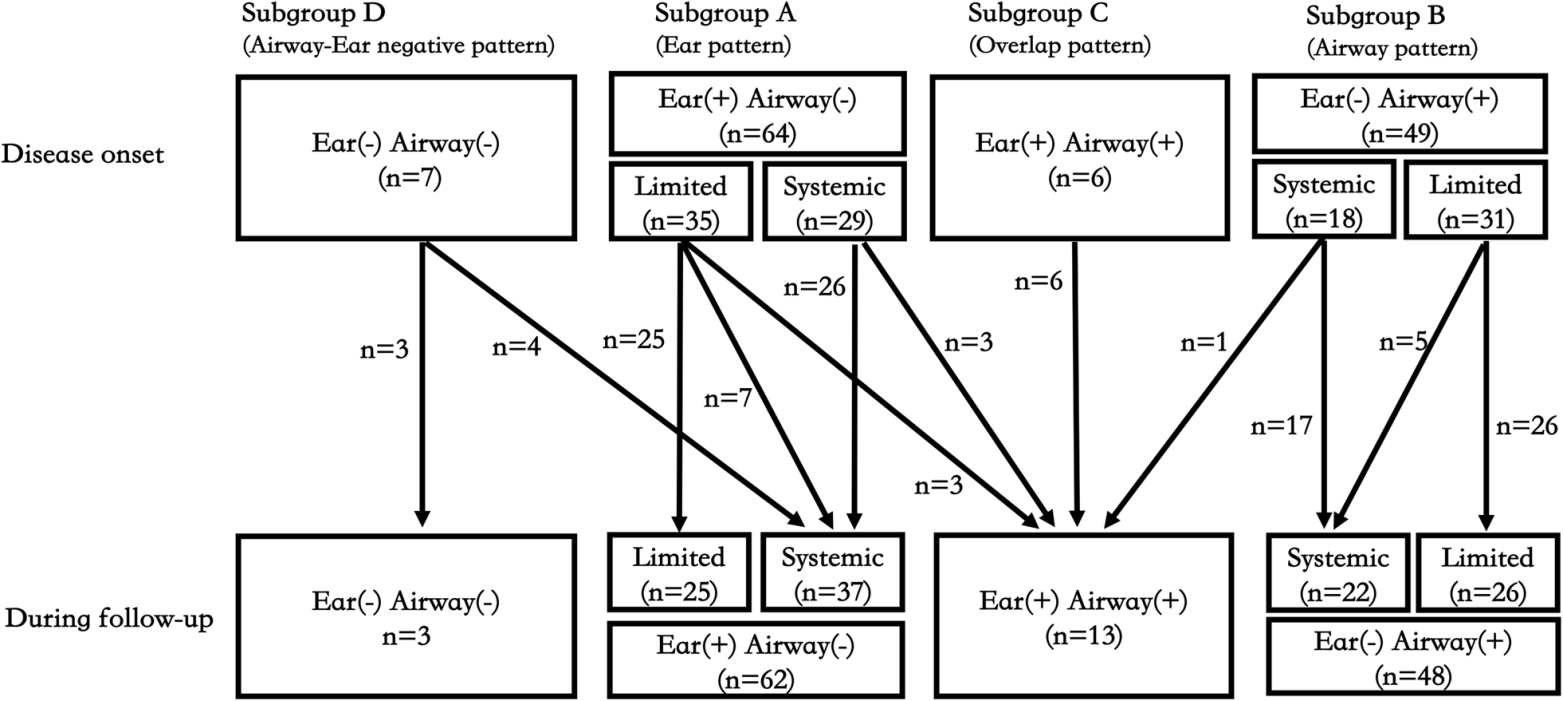
The evolution of clinical patterns and presenting forms from disease onset to last visit

We then analyzed the evolution of clinical patterns from disease onset to the last visit (Fig. 2). A few evolution courses were noticed. First, one clinical pattern may progress to another one. Six patients with Ear pattern (3 limited form and 3 systemic form) developed airway lesions and one patient with Airway pattern developed auricular chondritis, which were collectively classified as Overlap pattern. Four patients with Airway-Ear negative pattern developed auricular chondritis and progressed into Ear pattern. Second, limited RPC may become systemic. Seven limited RPC patients with Ear pattern and 5 limited RPC patients with Airway pattern progressed into systemic disease. Third, a large majority of the limited RPC patients (25 with Ear pattern and 26 with airway pattern) remained unchanged during follow-up, indicating no disease progression in these patients.

### Clinical features in different patterns of RPC

Compared with those with Airway pattern, RPC patients with Ear pattern had a higher incidence of ocular involvement (38.7% vs 8.3%, P < 0.001) and arthritis (27.4% vs 4.2%, P = 0.002), and a relatively lower incidence of nasal chondritis (6.5% vs 25%, P = 0.011). Of note, RPC patients with Airway pattern had higher mortality rate compared with those with Ear pattern (29.2% vs 1.6%, P = 0.015). Interestingly, Overlap pattern seems to be a combination of Ear pattern and Airway pattern as those patients had an intermediate rate of ocular inflammation (23.1%), arthritis (15.4%) and mortality (23.1%), between that of those with Ear pattern and Airway pattern, except a relatively higher incidence of hearing loss (23.1%) and nasal chondritis (30.8%) (Table 3). Among 3 patients with Airway-Ear negative pattern, all had ocular inflammation and 2 had hearing loss, nasal chondritis, and arthritis. No significant difference of ages at disease onset was detected between different patterns but Ear pattern presented lower CRP levels compared with Airway pattern and Overlap pattern, indicating relatively lower inflammatory status of these patients (Table 3).

**Table 3:**
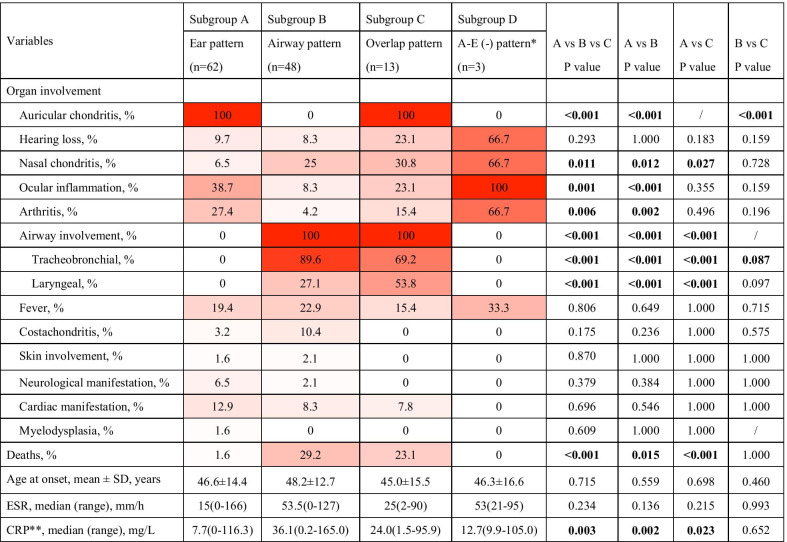
Different characteristics of 4 clinical patterns

Note: P-values in boldface indicate statistical significance (P < 0.05). Airway-Ear (-) pattern was not compared with other patterns, due to limited number of patients

^*^ A-E (-) pattern: Airway-Ear negative pattern

^**^CRP, C-reactive protein

There were 18 deaths (14.3%) during a median follow-up of 23.5 months (range 5–81 months), and the causes of deaths were refractory disease (n = 13), pulmonary infection (n = 3), brain tumor (n = 1), and unknown cause (n = 1). One patient was with ear pattern (died of brain tumor), 14 with airway pattern and 3 with overlap pattern.

**Fig. 3:**
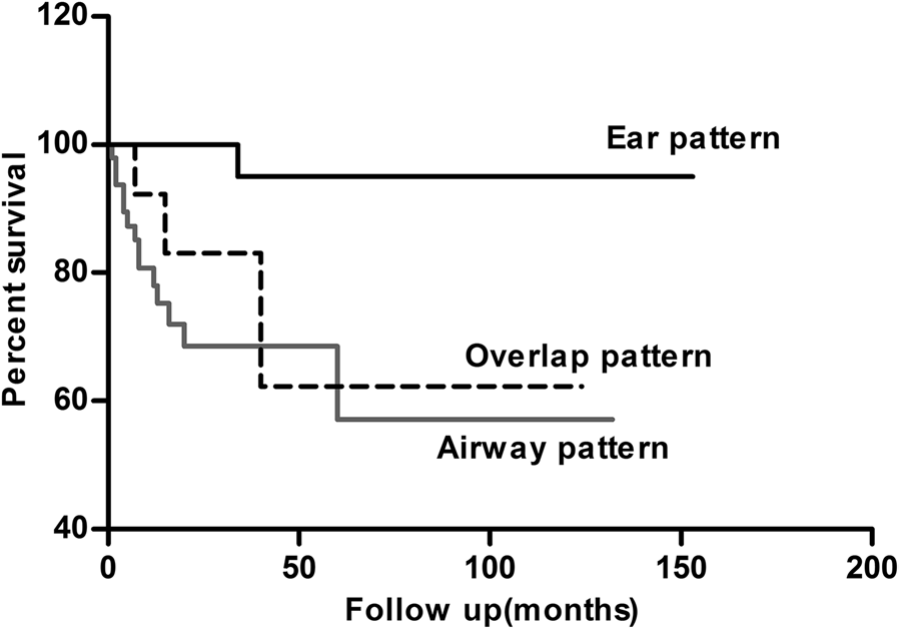
Survival curve of Ear pattern, Airway pattern and Overlap pattern

The probability of survival was significantly different between Ear pattern and the other 2 patterns, whereas no difference was detected between Airway pattern and Overlap pattern (Fig. 3), suggesting that airway involvement may be a predominant prognostic factor.

## Discussion

Early diagnosis of RPC remains an unmet medical need after its first description in 1932 despite the introduction of empirical diagnostic criteria by McAdams et al. [14], Damiani et al. [15] and Michet et al. [16]. In the absence of updated criteria, it is important for physicians, especially non-rheumatologic ones, to keep vigilance for this rare disease in order to make prompt diagnosis. To achieve this goal, detailed characterization of different presenting patterns is warranted in RPC patients. Herein, we report a retrospective cohort of 126 Chinese RPC patients with definition of 4 clinical patterns and 2 presenting forms (limited and systemic forms) and described the evolution of clinical patterns during follow-up.

Cumulative features of large case series were summarized in Table 1 and most series were about Caucasians. Clinical manifestations may differ in patients from different regions. The age at diagnosis of our patients was comparable to previous reports. The diagnostic delay was much shorter than previous study, indicating more RPC patients in early stage were included in our study. Comparable airway involvements but lower frequency of other features were observed, which may be due to the shorter follow-up period of our study. Meanwhile, it also demonstrated relatively more severe disease in Chinese population, since more patients in our cohort developed airway involvement in a relatively shorter follow-up duration compared with other reports. Consistent with Lin’s findings [7], the patients in our study had a higher initial frequency of laryngo-tracheal involvement (42.9%) than the Caucasians (14–38% initially), further demonstrating more severe disease in Chinese population [32].

Clinical classification is of vital importance to for early diagnosis. Dion et al. [27] and Shimizu et al. [28, 29] have made such attempts, however, these two studies did not cover all clinical spectrum of this disease. Dion and colleagues divided RPC into 3 clinical subtypes: hematologic (with the worst prognosis), respiratory and mild phenotype [27]. However, hematologic involvement constituents a relative larger part in this cohort (8% had MDS and 13% had hematologic diseases) than that in Chinese patients (only 3.2% in our cohort and no patient in Lin’s cohort had MDS), indicating that this classification is not suitable for Chinese patients. In addition, this classification seems not to be developed for early diagnosis since this classification was based on disease severity, progression and prognosis instead of the initial presentations. Correlation analysis of 239 cases by Shimizu et al. [28] revealed a negative correlation between airway involvement and external ear involvement (r = − 0.48). Therefore, the disease was divided into three clinical subgroups [29]: airway involvement (47 cases), external ear involvement (118 cases) and both airway and external ear involvement (70 cases). However, this study did not include limited RPC or partial RPC. The re-analysis of the cohort data by French researchers also found a negative correlation between tracheobronchial involvement and external ear involvement (r = − 0.245, P = 0.003) but not the airway overall [33].

Compared to the observations made in French and Japanese patients [28, 29, 34], our current study demonstrated a much stronger negative correlation between airway involvement and auricular chondritis (r = − 0.754, P < 0.001). Based on these findings, we divided RPC patients into 4 clinical patterns: Ear pattern (Subgroup A), Airway pattern (Subgroup B), Overlap pattern (Subgroup C) and Airway-Ear negative pattern (Subgroup D). The fact that patients with Ear pattern had the best prognosis and relatively lower inflammatory status, further suggests that patients with ear involvement can be a distinct clinical pattern. A large majority of patients had Ear pattern and Airway pattern, while the percentages of Overlap pattern and Airway-Ear negative pattern were relatively small. However, the clinical pattern did not remain unchanged during follow-up. Ear pattern and Airway pattern may progress into Overlap pattern and Airway-Ear negative pattern into ear pattern. The progression of the disease was also reflected by the fact that limited form may progress into systemic form. Particular attention should be paid to the patients with Ear pattern that progressed into Overlap pattern because devastating events may ensue. Clinical characterization of different patterns indicate that auricular chondritis is related to ocular inflammation and arthritis, and airway involvement is related to nasal chondritis, suggesting different pathognomonic mechanisms in different clinical patterns.

Patients with laryngo-tracheal involvement or auricular inflammation as the only initial feature of the disease [13], sometimes as the sole feature [8, 17–24], have been defined as limited form of RPC. In our cohort, 52.3% of the patients presented as limited RPC initially and 40.5% of them remained limited form during follow-up, suggesting that limited RPC is not uncommon among Chinese patients. The concept of limited and systemic form is also supported by a recent online survey in the US [8], but the incidence of limited RPC was much lower (16 and 2 out of 304 patients presented as isolated ear involvement and isolated airway involvement respectively).

There are some limitations of our study. First, this retrospective analysis of the hospitalized patients did not include patients in out-clinics, thus comprehensive and real scenario of RPC may not be well represented. The recall bias may also exist, but we called back most of our patients to confirm the history. Second, the relatively short follow-up duration may underestimate the frequency of organ involvements. However, our study also has some strengthens. First, we include RPC patients in early stage because the diagnostic delay was much shorter than previous study which may facilitate early recognition of RPC. Second, we included full spectrum of this rare disease because a proportion of our patients presented as partial RPC and limited RPC, which further strengthen the consensus that new classification criteria should be put on agenda.

## Conclusions

In conclusion, our study included early RPC patients, defined distinct characteristics of Chinese RPC patients and categorized them into 4 different clinical patterns and 2 distinct presenting forms (limited and systemic forms) which may facilitate early recognition of this rare disease. In addition, we believe our study may contribute to an updated classification criteria covering all the clinical spectrum of RPC.

## Methods

### Study population

We retrospectively reviewed the medical records of 126 RPC patients that were hospitalized and followed-up by rheumatologists at our hospital between January, 2008 and August, 2019. RPC was defined according to traditional criteria proposed by Michet et al. [16] and Damiani et al. [15]. Patients that underwent recurrent chondritis associated with deformity, vestibular dysfunction, ocular inflammation, or inflammatory arthritis were diagnosed as partial RPC as suggested by Mathew et al. [11]. Limited RPC can also be diagnosed if patients presented with recurrent inflammatory episodes at isolated cartilaginous sites after exclusion of other possible causes and are responsive to glucocorticoids, based on the observations by other groups and our own [6, 8, 13, 17–24]. The diagnostic criteria used in present study were listed in Table 4. Patients younger than 18 years or without complete electronic case files were excluded. Patients with positive anti-neutrophilic cytoplasmic antibody (ANCA) against proteinase-3 (PR3) were also excluded as suggested by Piette and colleagues [34]. The testing of ANCA was performed using Euroimmun AG detecting system (Lübeck, Germany), according to the procedures suggested by the instruction. The diagnosis was carefully evaluated and jointly established by a group of rheumatologists and physicians of other specialties. The recorded clinical data included manifestations associated with external ear, inner ear, nose, larynx, joints, costochondral cartilage, tracheobronchial tree, eye, heart, skin, and central nervous system involvement, as well as constitutional symptoms.

**Table 4:** Diagnostic criteria of relapsing polychondritis used in present study

Criteria	Items of criteria	Requirement
McAdam’s criteria [14]	Bilateral auricular chondritis Nasal chondritis Respiratory tract chondritis Non-erosive seronegative polyarthritis Ocular inflammation Cochlear and/or vestibular dysfunction	3 out of 6 criteria
Damiani’s criteria [15]	3 of 6 McAdam et al. criteria 1 of 6 McAdam et al. criteria + histologic confirmation 2of 6 McAdam et al. criteria + Response to corticosteroids or dapsone	Any of these
Michet’s criteria [16]	Major criteria: Auricular cartilage inflammation Nasal cartilage inflammation Laryngotracheal cartilage inflammation Minor criteria: Ocular inflammation Hearing loss Vestibulary dysfunction Seronegative arthritis	2 major criteria or 1 major criteria + 2 minor criteria
Criteria of Partial RPC [11]	A. Recurrent chondritis with deformity B. Vestibular dysfunction C. Ocular inflammation D. Inflammatory arthritis	A + any of B to D
Criteria of Limited RPC [6, 8, 13, 17–24]	A. Recurrent inflammatory episodes at isolated cartilaginous sites B. Exclusion of other possible causes C. Responsive to glucocorticoids	A + B + C

### Definition of organ involvement

Auricular chondritis was defined as redness and swelling of the pinna accompanied with tenderness or cauliflower ear, sparing the lobule. Airway involvement include laryngeal and tracheobronchial chondritis. Laryngeal chondritis was defined by the symptoms of hoarseness and aphonia and should be confirmed by direct laryngoscopy, bronchoscopy or cervical CT scan showing swelling and narrowing of the larynx, including subglottis. Tracheobronchial chondritis was defined by thickness of anterior tracheal wall or narrowing of the trachea or bronchi, visualized by CT scan or bronchoscopy, with or without calcification of the tracheobronchial wall. Nasal chondritis was defined as documented nasal pain with redness and swelling or saddle nose. Ocular inflammation was defined as physician-observed scleritis, episcleritis, iritis or uveitis. Arthritis was defined as documented joint pain and swelling. Hearing loss was defined acute-onset deafness that may not be explained by other causes. Costochondritis was defined by documented pain and/or tenderness of at least one costo-sternal joint. Cardiac involvement include arrhythmia detected by electrocardiogram (ECG), valulopathy detected by echocardiogram and myocarditis confirmed by cardiac enzymes and/or ECG changes without other causes. Neurological involvement included encephalitis, cerebral vasculitis and psychiatric symptoms due to inflammation of the brain parenchyma confirmed by MRI and CSF, without other causes. Fever was defined as elevated body temperature documented in case files after exclusion of other causes. Cutaneous manifestation includes purpura or eruptions without other causes. Diagnosis of myelodysplastic syndrome (MDS) was established by a hematologist based on bone marrow examination.

### Statistical analysis

Descriptive statistics were used to describe demographic and disease characteristics, and all results were expressed as mean ± standard deviation (SD), median (range) or percentage (%) where appropriate. Continuous variables were compared using Student’s t-test where the data had normal distribution or Wilcoxon rank sum test where the data were not normally distributed. Categorical variables were compared using Fisher’s exact test or chi-square test, if appropriate.

The numbers 1 and 0 were assigned to describe the presence or absence, respectively, of each organ involvement, just as previous study [28, 29, 33]. Then correlation analysis was performed to disclose the correlation between organ involvement and correlation coefficients were calculated. A correlation coefficient r > 0 represents positive correlation and r < 0 represents negative correlation. When doing correlation analysis, the cumulative organ involvement was incorporated. Cluster analysis was performed with the method of hierarchical cluster. Survival rates over time were plotted using the Kaplan–Meier method and compared by log rank test. A two-sided P-value < 0.05 was considered to be statistically significant. Statistical analyses were performed using the SPSS version 17.0 software package (IBM).

## Data Availability

Not applicable.

## Abbreviations

RPC: Relapsing polychondritis
ANCA: Anti-neutrophilic cytoplasmic antibody
CRP: C-reactive protein
